# Supplementary material for: The splenic T cell receptor repertoire during an immune response against a complex antigen: Expanding private clones accumulate in the high and low copy number region
Source: PLoS One. 2022 Aug 24;17(8):e0273264. doi: 10.1371/journal.pone.0273264 (PMC9401120; doi:10.1371/journal.pone.0273264)
Supplement: S1 Fig — (PDF) [file pone.0273264.s001.pdf]

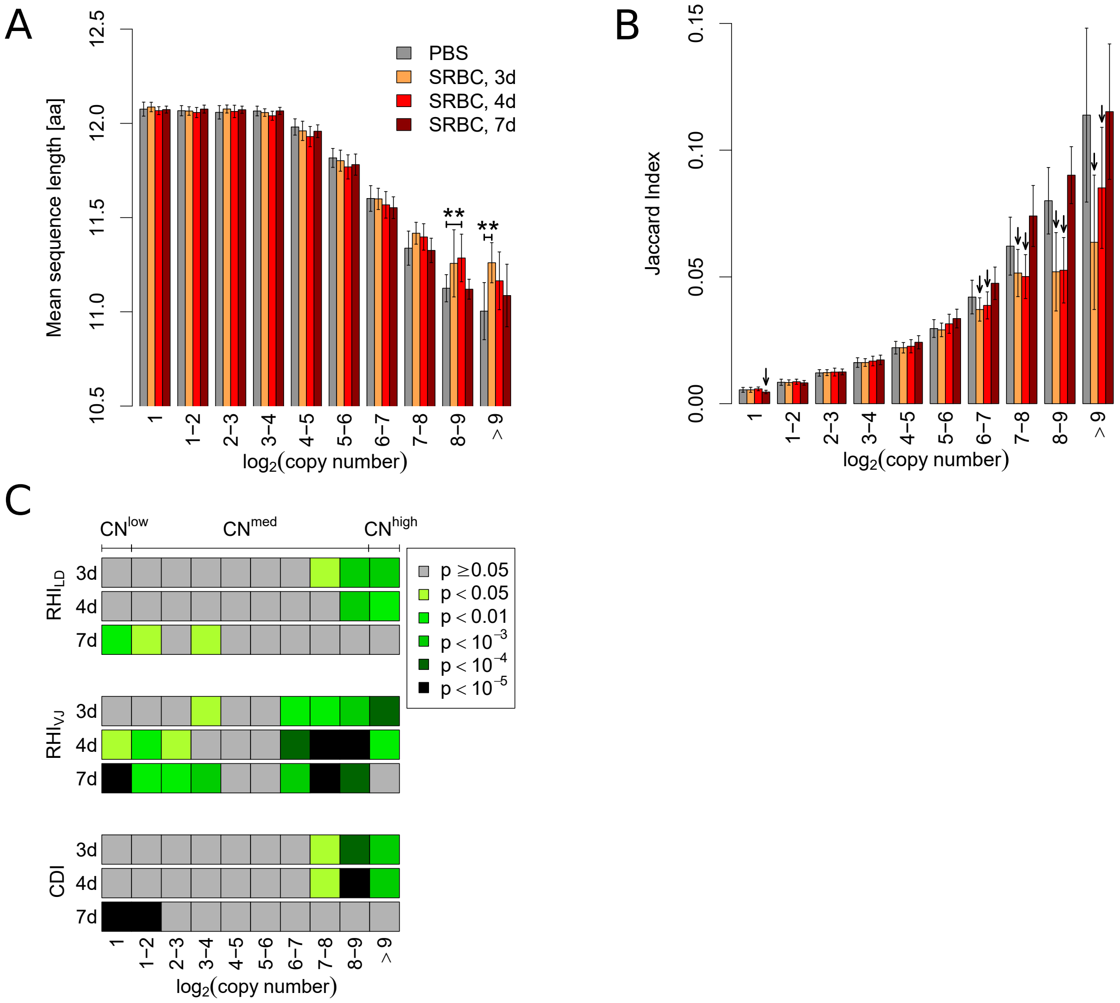

S1 Fig. Logarithmic fractioning of the repertoire shows graduated shifting of certain parameters with the intermediate part of the repertoire untouched by immunization-induced effects. Total repertoires of control (PBS-injected) animals (n=20) and mice 3, 4, and 7 days (d) after immunization with SRBC (n=10 each) where split into 10 fractions according to their copy number based on the logarithm to base 2. (A) Mean CDR3β sequence length and (B) clonal overlap measured by the Jaccard Index. Bars and whiskers display means and standard deviations. For (A) immunized repertoires were tested for deviations from control using the Mann-Whitney-U-test with p-values displayed as ** $p < 0.01$. Correction for multiple testing was performed using Holm's method. For (B) apparent immunization effects are highlighted by arrows. (C) For each fraction and time point immunized repertoires were compared to control for three parameters: the Repertoire Homogeneity Index (RHI) assessing repertoire homogeneity either i) concerning CDR3β sequence similarity measured by the Levenshtein distance (RHI_{LD}) or ii) VJ segment usage (RHI_{VJ}) as well as iii) the Coding Diversity Index (CDI) assessing heterogeneity of clonotype coding. Significant deviations indicate a decrease of the corresponding parameter. In difference to the corresponding figure in the main text (Fig 2B), p-values were not corrected for multiple testing to demonstrate that the lack of immunization-induced effects in the medium region of the repertoire ($4 < \log_2(CN) \leq 6$) as well as in the top fraction 7d after immunization was not caused by correction steps.
