# Supplementary material for: The splenic T cell receptor repertoire during an immune response against a complex antigen: Expanding private clones accumulate in the high and low copy number region
Source: PLoS One. 2022 Aug 24;17(8):e0273264. doi: 10.1371/journal.pone.0273264 (PMC9401120; doi:10.1371/journal.pone.0273264)
Supplement: S2 Fig — (PDF) [file pone.0273264.s002.pdf]

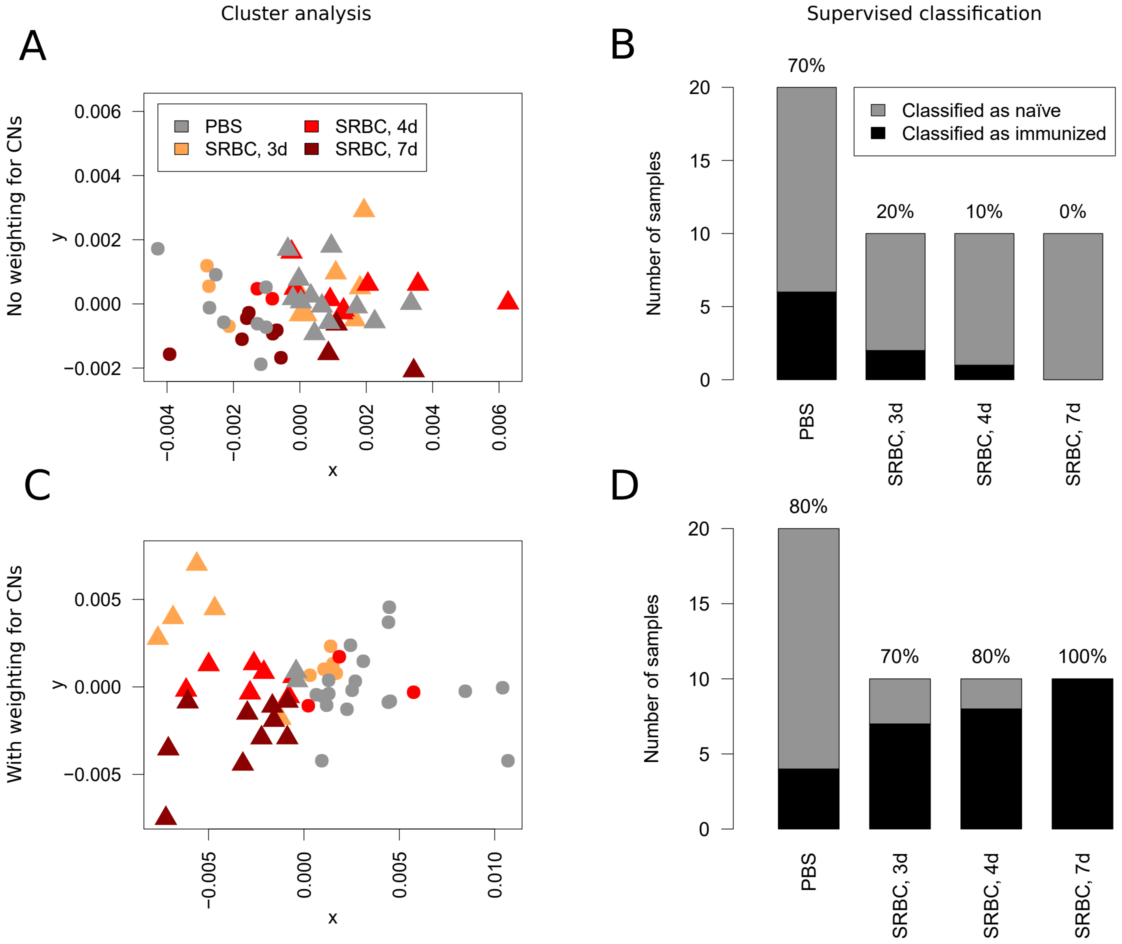

S2 Fig. Classification of total repertoires is successful only when weighted for copy numbers. (A) A dissimilarity matrix was calculated based on VJ segment usage (see Material and Methods for details) and applied to total repertoires of control (PBS-injected) animals and animals 3, 4 and 7 days (d) after immunization with SRBC neglecting copy numbers of clonotypes. When data were divided into two clusters using the K-medoid algorithm (result visualized via metric multidimensional scaling with dissimilarities approximated by distances of points in the scatterplot and clusters defined by the algorithm displayed as circles and triangles, respectively) none of the experimental groups formed a separate cluster. (B) The same dissimilarity matrix was used for a supervised classification of the same data sets, with the numbers on top of each bar denoting the percentage of correct classified samples indicating that most data sets were misclassified. (C)-(D) Weighting the clonotypes by copy number (see S1 Methods) led to distinct improvement of the classification results of total repertoires with (C) little overlap between the control and immunized cluster and (D) correct classification of 41 out of the 50 samples in total.
