# Supplementary material for: The splenic T cell receptor repertoire during an immune response against a complex antigen: Expanding private clones accumulate in the high and low copy number region
Source: PLoS One. 2022 Aug 24;17(8):e0273264. doi: 10.1371/journal.pone.0273264 (PMC9401120; doi:10.1371/journal.pone.0273264)
Supplement: S1 Methods — For statistic properties, which differ from those of the original versions, detailed analytic proofs are provided. Furthermore, we provide weighted versions (counting for copy numbers) for two of these indices. (PDF) [file pone.0273264.s003.pdf]

Supplemental Methods

In the following we establish some elementary properties of the indices which we applied in our study. For simplicity, we restrict our considerations to the analysis of the β-chain of T cell receptors (TCRs), although these indices are in fact not restricted to this purpose.

Let Ω denote the set of all possible TCRβ-chains. A data set X of such sequences can be interpreted as a finite set of pairs $\big(x_i, \nu_X(x_i)\big)_{i=1,\dots,m}$, where $\nu_X(x)$ denotes the copy number (CN) of a sequence x in X. For convenience, we use the term X synonymously for the data set and for the corresponding set of TCRβ-chains. Let us first have a look at three statistic indices which have originally been developed for ecological issues, but are also commonly used for the analysis of the T cell receptor repertoire (TCR-R).

Definition 1. *Let* $X = \big(x_i, \nu_X(x_i)\big)_{i=1,\dots,m}$ *and* $Y = \big(y_i, \nu_Y(y_i)\big)_{i=1,\dots,n}$ *be two arbitrary CDR3β data sets where the number of read sequences (including repeats) is given by* $M = \sum_{i=1}^{m} \nu_X(x_i)$ *and* $N = \sum_{i=1}^{n} \nu_Y(y_i)$ *respectively. We denote by*

(i)

$$D(X) = 1 - \frac{1}{M(M-1)} \sum_{i=1}^{m} \nu_X(x_i) \cdot (\nu_X(x_i) - 1)$$

the Simpson Index*, by*

(ii)

$$\mathrm{MH}(X,Y) = \frac{2 \sum_{z \in X \cap Y} \nu_X(z) \nu_Y(z)}{N \cdot M \Big(\sum_{x \in X} \frac{\nu_X(x)^2}{M^2} + \sum_{y \in Y} \frac{\nu_Y(y)^2}{N^2} \Big)}$$

the Morisita-Horn Index *and by*

(iii)

$$S(X,Y) = \frac{2\,|X \cap Y|}{|X| + |Y|}$$

the Sørensen Index*.*

Remark 1.

- *If two sequences are drawn from X with consideration of replications (i.e. CNs) and without replacement, $D(X)$ gives the probability of drawing two different sequences. So D is a measure for the diversity of X.*

- *The Morisita-Horn Index* $\mathrm{MH}(X,Y)$ *is a measure for the similarity of the data sets X and Y. It is equal to zero if and only if the data sets are disjoint and it is equal to one if and only if the two data sets consist of equal sequences where the percentage of each sequence in the total of the reads in the data sets is equal, i.e. if*

$$\frac{\nu_X(z)}{M} = \frac{\nu_Y(z)}{N} \qquad \forall z \in X \cup Y$$

- *The Sørensen Index is also a measure for the similarity of the data sets. It is equal to zero if the data sets (i.e. the corresponding clone sets) are disjoint and it is equal to one if the sets coincide. In difference to the Morisita-Horn Index the CNs are neglected.*

These indices can be generalized in a straight forward manner by considering an arbitrary criterion of similarity instead of equality. In the following we denote by

- $R \subset \Omega \times \Omega$ an arbitrary reflexive, symmetric relation on Ω and by
- $\mathbb{1}(\cdot)$ the indicator function.

Definition 2. *Let $X = \big(x_i, \nu_X(x_i)\big)_{i=1,\ldots,m}$ be an arbitrary data set of TCRβ sequences where the total number of read sequences (including repeats) is given by $M = \sum_{i=1}^{m} \nu_X(x_i)$. In dependence of the relation R we define*

- *the* weighted Repertoire Homogeneity Index *as*

$$\mathrm{wRHI}(X) = \frac{1}{M\,(M-1)} \sum_{i,j=1,\ldots,m} \nu_X\,(x_i)\,\big(\nu_X\,(x_j) - \mathbb{1}(i=j)\big)\mathbb{1}(x_i R x_j)$$

- *and the unweighted analogue as the* Repertoire Homogeneity Index *which is given by*

$$\mathrm{RHI}(X) = \frac{\displaystyle\sum_{\substack{i=1,\ldots,m\\ j<i}} \mathbb{1}(x_i R x_j)}{\binom{m}{2}}$$

Remark 2. $\mathrm{wRHI}(X)$ *can be interpreted as the probability that two randomly drawn sequences of X represent clones which are related in respect of R.* $\mathrm{RHI}(X)$ *can be derived from* $\mathrm{wRHI}(X)$ *by neglecting the CNs. We directly obtain the following properties:*

- wRHI, RHI $\in [0,1]$,
- $\mathrm{RHI}(X) = 0 \Leftrightarrow \neg(x_i R x_j) \qquad \forall i,j = 1,\ldots,m,\ i \neq j,$
- $\mathrm{wRHI}(X) = 1 \Leftrightarrow \mathrm{RHI}(X) = 1 \Leftrightarrow x_i R x_j \qquad \forall i,j = 1,\ldots,m,$
- *if R denotes the identity relation we obtain*

$$\mathrm{wRHI}(X) = 1 - D(X).$$

For the comparison of two different data sets, we define two further indices.

Definition 3. *Let $X = \big(x_i, \nu_X(x_i)\big)_{i=1,\ldots,m}$ and $Y = \big(y_i, \nu_Y(y_i)\big)_{i=1,\ldots,n}$ be arbitrary data sets of TCRβ sequences, where the total number of read sequences (including repeats) is given by $M = \sum_{i=1}^{m} \nu_X(x_i)$ and $N = \sum_{i=1}^{n} \nu_Y(y_i)$, respectively. In dependence of the relation R we define*

- *the* weighted Repertoire Similarity Index *of* X *and* Y *as*

$$\mathrm{wRSI}_R(X,Y) = \frac{2\sum\limits_{\substack{i=1,\dots,m\\ j=1,\dots,n}} \nu_X(x_i)\nu_Y(y_j)\mathbb{1}(x_iRy_j)}{NM\Bigg(\sum\limits_{i,j=1,\dots,m} \frac{\mathbb{1}(x_iRx_j)\nu_X(x_i)\nu_X(x_j)}{M^2} + \sum\limits_{i,j=1,\dots,n} \frac{\mathbb{1}(y_iRy_j)\nu_Y(y_i)\nu_Y(y_j)}{N^2}\Bigg)}$$

- *and the unweighted analogue as the* Repertoire Similarity Index *of* X *and* Y *which is given by*

$$\mathrm{RSI}_R(X,Y) = \frac{2\sum\limits_{\substack{i=1,\dots,m\\ j=1,\dots,n}} \mathbb{1}(x_iRy_j)}{nm\Bigg(\sum\limits_{i,j=1,\dots,m} \frac{\mathbb{1}(x_iRx_j)}{m^2} + \sum\limits_{i,j=1,\dots,n} \frac{\mathbb{1}(y_iRy_j)}{n^2}\Bigg)}.$$

Proposition 1.

(i) wRSI, RSI ≥ 0.

(ii) If R *denotes the identity relation the two indices are given by*

$$\mathrm{wRSI}_R(X,Y) = \mathrm{MH}(X,Y) \quad \text{and} \quad \mathrm{RSI}_R(X,Y) = S(X,Y).$$

(iii) The relation R *is transitive (i.e. an equivalence relation) if and only if* 1 *is an upper bound for one (and with that for both) of the two indices, i.e. if*

$$\mathrm{wRSI}_R\,,\ \mathrm{RSI}_R \leq 1$$

for all pairs of data sets X *and* Y*.*

(iv) If

$$\frac{\sum\limits_{i=1}^{m} \nu_X(x_i)\mathbb{1}(x_iRz)}{M} = \frac{\sum\limits_{i=1}^{n} \nu_Y(y_i)\mathbb{1}(y_iRz)}{N} \qquad \forall z \in X \cup Y \tag{1}$$

then $\mathrm{wRSI}_R(X,Y) = 1$*. The opposite direction is also valid if* R *is transitive.*

(v) An analogous result to (iv) is obtained for the Repertoire Similarity Index. If

$$\frac{\sum\limits_{i=1}^{m} \mathbb{1}(x_iRz)}{m} = \frac{\sum\limits_{i=1}^{n} \mathbb{1}(y_iRz)}{n} \qquad \forall z \in X \cup Y$$

then $\mathrm{RSI}_R(X,Y) = 1$*. The opposite direction is also valid if* R *is transitive.*

Proof. We only show the statements concerning wRSI_R. For RSI_R the proof can be performed analogously by assigning a dummy copy number of 1 to each clone. (i) and (ii) are trivial. To validate (iii) let us first assume that R is transitive, i.e. an equivalence relation. This implies that there exists two sets of equivalence classes $\left(C(x_i')\right)_{i=1,\ldots,m'}$ and $\left(C(y_i')\right)_{i=1,\ldots,n'}$ satisfying

$$X = \bigcup_{i=1}^{m'} C(x_i'), \qquad Y = \bigcup_{i=1}^{n'} C(y_i')$$

and

$$C(x_i') \cap C(x_j') = \emptyset \quad \forall i,j = 1,\ldots,m',\, i \neq j,$$
$$C(y_i') \cap C(y_j') = \emptyset \quad \forall i,j = 1,\ldots,n',\, i \neq j,$$

where the sequences $(x_i')_{i=1,\ldots,m'}$ and $(y_i')_{i=1,\ldots,n'}$ are arbitrary representatives of the corresponding equivalence classes. We denote by

$$M(x_k') = \sum_{i=1}^{m} \nu_X(x_i) \mathbb{1}\left(x_i R x_k'\right), \quad k = 1,\ldots,m' \quad \text{and}$$
$$N(y_k') = \sum_{i=1}^{m} \nu_Y(y_i) \mathbb{1}\left(y_i R y_k'\right), \quad k = 1,\ldots,n'$$

the summated reads of the clones in the corresponding equivalence class. If there is no sequence in X which is related to any sequence in Y (and vice versa) the claim is trivial. Otherwise we may assume without loss of generality that there exists $s \in \mathbb{N}$, such that

$$x_i' R y_j' \Leftrightarrow i, j \leq s \wedge i = j.$$

Otherwise the indexing of representatives could be modified. We obtain

$$\begin{aligned} 0 &\leq \sum_{i=1}^{s} \left(\frac{M(x_i')}{M} - \frac{N(y_i')}{N} \right)^2 \\ &= \sum_{i=1}^{s} \frac{M(x_i')^2}{M^2} - \sum_{i=1}^{s} \frac{2M(x_i')N(y_i')}{MN} + \sum_{i=1}^{s} \frac{N(y_i')^2}{N^2}. \end{aligned}$$

Adding the negative summand to both sides of the inequality yields

$$\frac{2}{NM} \sum_{i=1}^{s} M(x_i')N(y_i') \leq \sum_{i=1}^{s} \frac{M(x_i')^2}{M^2} + \sum_{i=1}^{s} \frac{N(y_i')^2}{N^2}.$$

This implies

$$
\begin{aligned}
\frac{2}{MN}\sum_{i=1}^{s} M(x_i')N(y_i') &= \frac{2}{MN}\sum_{k=1}^{s}\Big(\sum_{i=1}^{m}\nu_X(x_i)\mathbb{1}\left(x_iRx_k'\right)\sum_{i=1}^{n}\nu_Y(y_i)\mathbb{1}\left(y_iRy_k'\right)\Big) \\
&= \frac{2}{MN}\sum_{k=1}^{s}\sum_{\substack{i=1,\ldots,m\\ j=1,\ldots,n}}\nu_X(x_i)\nu_Y(y_j)\mathbb{1}(x_iRx_k' \wedge y_jRy_k') \\
&= \frac{2}{MN}\sum_{\substack{i=1,\ldots,m\\ j=1,\ldots,n}}\nu_X(x_i)\nu_Y(y_j)\mathbb{1}(x_iRy_j) \\
&\leq \sum_{i=1}^{s}\frac{M(x_i')^2}{M^2} + \sum_{i=1}^{s}\frac{N(y_i')^2}{N^2} \\
&\leq \sum_{i=1}^{m'}\frac{M(x_i')^2}{M^2} + \sum_{i=1}^{n'}\frac{N(y_i')^2}{N^2} \\
&= \sum_{k=1}^{m'}\frac{1}{M^2}\Big(\sum_{i=1}^{m}\nu_X(x_i)\mathbb{1}(x_iRx_k')\Big)^2 \\
&+ \sum_{k=1}^{n'}\frac{1}{N^2}\Big(\sum_{i=1}^{n}\nu_Y(y_i)\mathbb{1}(y_iRy_k')\Big)^2 \\
&= \sum_{k=1}^{m'}\frac{1}{M^2}\sum_{i,j=1,\ldots,m}\nu_X(x_i)\nu_X(x_j)\mathbb{1}(x_iRx_k' \wedge x_jRx_k') \\
&+ \sum_{k=1}^{n'}\frac{1}{N^2}\sum_{i,j=1,\ldots,n}\nu_Y(y_i)\nu_Y(y_j)\mathbb{1}(y_iRy_k' \wedge y_jRy_k') \\
&= \sum_{i,j=1,\ldots,m}\frac{\nu_X(x_i)\nu_X(x_j)\mathbb{1}(x_iRx_j)}{M^2} \\
&+ \sum_{i,j=1,\ldots,n}\frac{\nu_Y(y_i)\nu_Y(y_j)\mathbb{1}(y_iRy_j)}{N^2}.
\end{aligned}
$$

In summary we obtain

$$\frac{2}{MN}\sum_{\substack{i=1,\dots,m\\ j=1,\dots,n}}\nu_X(x_i)\nu_Y(y_j)\mathbb{1}(x_iRy_j) \le \sum_{i,j=1,\dots,m}\frac{\nu_X(x_i)\nu_X(x_j)\mathbb{1}(x_iRx_j)}{M^2} + \sum_{i,j=1,\dots,n}\frac{\nu_Y(y_i)\nu_Y(y_j)\mathbb{1}(y_iRy_j)}{N^2}.$$

Dividing the inequality by the right term yields the claim.

If R is non-transitive there exists at least one triplet a, b, c of sequences satisfying

$$aRb, \quad bRc \quad \text{and} \quad \neg(aRc). \tag{2}$$

We define two dummy data sets

$$X = \{(a,1),\ (c,1)\} \quad \text{and} \quad Y = \{(b,1)\} \tag{3}$$

and obtain

$$\text{wRSI}_R(X,Y) = \frac{4}{3} > 1.$$

It remains to verify (iv). Let us first assume that (1) is fulfilled. The formula for the weighted Repertoire Similarity Index can be written as

$$\begin{aligned}\text{wRSI}_R(X,Y) &= \frac{\frac{2}{MN}\sum\limits_{\substack{i=1,\dots,m\\ j=1,\dots,n}}\nu_X(x_i)\nu_Y(y_j)\mathbb{1}(x_iRy_j)}{\sum\limits_{i=1}^{m}\sum\limits_{j=1}^{m}\frac{\nu_X(x_i)\nu_X(x_j)\mathbb{1}(x_iRx_j)}{M^2}+\sum\limits_{i=1}^{n}\sum\limits_{j=1}^{n}\frac{\nu_Y(y_i)\nu_Y(y_j)\mathbb{1}(y_iRy_j)}{N^2}} \\ &= \frac{\frac{2}{MN}\sum\limits_{\substack{i=1,\dots,m\\ j=1,\dots,n}}\nu_X(x_i)\nu_Y(y_j)\mathbb{1}(x_iRy_j)}{\sum\limits_{i=1}^{m}\left(\frac{\nu_X(x_i)}{M}\sum\limits_{j=1}^{m}\frac{\nu_X(x_j)\mathbb{1}(x_iRx_j)}{M}\right)+\sum\limits_{i=1}^{n}\left(\frac{\nu_Y(y_i)}{N}\sum\limits_{j=1}^{n}\frac{\nu_Y(y_j)\mathbb{1}(y_iRy_j)}{N}\right)}.\end{aligned}$$

Inserting (1) in both summands of the denominator yields

$$\begin{aligned}\text{wRSI}_R(X,Y) &= \frac{\frac{2}{MN}\sum\limits_{\substack{i=1,\dots,m\\ j=1,\dots,n}}\nu_X(x_i)\nu_Y(y_j)\mathbb{1}(x_iRy_j)}{\frac{1}{MN}\left(\sum\limits_{i=1}^{m}\sum\limits_{j=1}^{n}\nu_X(x_i)\nu_Y(y_j)\mathbb{1}(x_iRy_j)+\sum\limits_{i=1}^{n}\sum\limits_{j=1}^{m}\nu_Y(y_i)\nu_X(x_j)\mathbb{1}(x_jRy_i)\right)} \\ &= 1.\end{aligned}$$

For the opposite direction we assume that R is transitive and $\mathrm{wRSI}_R(X,Y)=1$. This implies that

$$\frac{2\sum\limits_{\substack{i=1,\dots,m\\ j=1,\dots,n}} \nu_X(x_i)\nu_Y(y_j)\mathbb{1}(x_iRy_j)}{MN} = \sum_{i,j=1,\dots,m}\frac{\nu_X(x_i)\nu_X(x_j)\mathbb{1}(x_iRx_j)}{M^2} + \sum_{i,j=1,\dots,n}\frac{\nu_Y(y_i)\nu_Y(y_j)\mathbb{1}(y_iRy_j)}{N^2}.$$

Using the notation introduced in the proof of (iii), this equation can be written as

$$2\sum_{i=1}^{s}\left(\frac{M(x_i')}{M}\cdot\frac{N(y_i')}{N}\right) = \sum_{i=1}^{s}\left(\frac{M(x_i')}{M}\right)^2 + \sum_{i=1}^{s}\left(\frac{N(y_i')}{N}\right)^2 + \sum_{i=s+1}^{m'}\left(\frac{M(x_i')}{M}\right)^2 + \sum_{i=s+1}^{n'}\left(\frac{N(y_i')}{N}\right)^2.$$

This can be rearranged as

$$-\sum_{i=1}^{s}\left(\frac{M(x_i')}{M}-\frac{N(x_i')}{N}\right)^2 = \sum_{i=s+1}^{m'}\left(\frac{M(x_i')}{M}\right)^2 + \sum_{i=s+1}^{n'}\left(\frac{N(y_i')}{N}\right)^2. \tag{4}$$

Since the left side of (4) is negative and the right side is positive we conclude that both sides are equal to zero. This implies that

$$M(x_i')=0 \quad \mathit{and} \quad N(y_j')=0 \quad \forall i,j>s$$

and

$$\frac{M(x_i')}{M}=\frac{N(y_i')}{N} \qquad \forall i=1,\dots,s.$$

Since for an arbitrary $z\in X\cup Y$ there exists exactly one $i\in\mathbb{N}$ satisfying $x_i'Rz$ this yields the claim. To demonstrate that transitivity is indeed required for this direction, we assume that R is non-transitive. For a triple fulfilling (2) we consider the two dummy data sets

$$X=\{(a,1),\ (c,1)\} \quad \text{and} \quad Y=\{(a,1),\ (b,1)\}.$$

The reader can easily verify that if z is set to a, (1) is violated although $\mathrm{wRSI}_R(X,Y)=1$. □

Remark 3. *For k different relations $R_1, \ldots, R_k$ attributed with appropriate weights $\alpha = (\alpha_1, \ldots, \alpha_k)$ satisfying $\alpha_i \geq 0$, $i = 1, \ldots, k$ and $\sum_{i=1}^{k} \alpha_i = 1$. The indices*

- $$d_{w,\alpha,R_1,\ldots,R_k}(X,Y) = 1 - \sum_{i=1}^{k} \alpha_i \min\big(\, \mathrm{wRSI}_{R_i}(X,Y), 1\big)$$

- $$d_{\alpha,R_1,\ldots,R_k}(X,Y) = 1 - \sum_{i=1}^{k} \alpha_i \min\big(\, \mathrm{RSI}_{R_i}(X,Y), 1\big)$$

provide a measure of dissimilarity of the data sets X and Y defined by the respective k criteria. We applied these indices for several classification experiments (see main text).
