# Supplementary material for: The splenic T cell receptor repertoire during an immune response against a complex antigen: Expanding private clones accumulate in the high and low copy number region
Source: PLoS One. 2022 Aug 24;17(8):e0273264. doi: 10.1371/journal.pone.0273264 (PMC9401120; doi:10.1371/journal.pone.0273264)
Supplement: S1 Table — (PDF) [file pone.0273264.s004.pdf]

Mouse	Group	Number of clonotypes	Number of reads
1	PBS	74756	1622167
2	PBS	83240	1562913
3	PBS	62906	1360119
4	PBS	59706	1401147
5	PBS	47433	1560822
6	PBS	81781	1969965
7	PBS	68248	1710775
8	PBS	86956	1403781
9	PBS	63620	1602940
10	PBS	71166	1989399
11	PBS	59646	1443388
12	PBS	74518	1348790
13	PBS	71206	2072181
14	PBS	56886	1425614
15	PBS	80036	1748015
16	PBS	70863	1565632
17	PBS	75941	1742824
18	PBS	71724	1529478
19	PBS	57351	1797106
20	PBS	57122	1296599
21	SRBC, 3d	63619	1746257
22	SRBC, 3d	70813	1465103
23	SRBC, 3d	65722	1852002
24	SRBC, 3d	60631	1586800
25	SRBC, 3d	72263	1892355
26	SRBC, 3d	88879	1760717
27	SRBC, 3d	82210	2118503
28	SRBC, 3d	64088	1526616
29	SRBC, 3d	59969	1953012
30	SRBC, 3d	67560	1924356
31	SRBC, 4d	78134	1731751
32	SRBC, 4d	54298	1234034
33	SRBC, 4d	62651	1505636
34	SRBC, 4d	71688	1407571
35	SRBC, 4d	59428	1943909
36	SRBC, 4d	67621	1743728
37	SRBC, 4d	62057	1117124
38	SRBC, 4d	81712	1347773
39	SRBC, 4d	54656	1537101
40	SRBC, 4d	56469	1316738
41	SRBC, 7d	70163	1589991
42	SRBC, 7d	86487	1585460
43	SRBC, 7d	62315	1592715
44	SRBC, 7d	77649	1587386
45	SRBC, 7d	72268	1589748
46	SRBC, 7d	62001	1592174
47	SRBC, 7d	66690	1591266
48	SRBC, 7d	76255	1588870
49	SRBC, 7d	70148	1590520
50	SRBC, 7d	96852	1581217

S1 Table. Number of extracted clonotypes and sequence reads of the 50 data sets after all preprocessing steps (including downsampling of the 7d group and removing clonotypes with CN 1).
