# Supplementary material for: The splenic T cell receptor repertoire during an immune response against a complex antigen: Expanding private clones accumulate in the high and low copy number region
Source: PLoS One. 2022 Aug 24;17(8):e0273264. doi: 10.1371/journal.pone.0273264 (PMC9401120; doi:10.1371/journal.pone.0273264)
Supplement: Attachment — Submitted filename: Response to Reviewers.pdf [file pone.0273264.s006.pdf]

UNIVERSITÄT ZU LÜBECK
INSTITUT FÜR ANATOMIE

Institut of Anatomy – Ratzeburger Allee 160 – 23562 Lübeck

Jörg Hermann Fritz, Ph.D

Academic Editor

PLOS ONE

Prof. Dr. med Jürgen Westermann

Ratzeburger Allee 160, 23562 Lübeck

Tel: +4945131017100

Fax: +4945131017104

E-Mail: juergen.westermann@uni-luebeck.de

Dear Dr. Fritz,

Thank you for your e-mail regarding our manuscript **"Tracking a diverse immune response against a complex cellular antigen in the murine T cell receptor repertoire: Over time, expanding private clones accumulate in the high but also the low copy number region"** (PONE-D-22-11828).

The comments of the reviewers have been very helpful. We revised the manuscript according to their suggestions and comment the changes in the following:

Reviewer 1

The term "tracking" is used in the manuscript title. The way I see it, this would imply the tracking of specific clonotypes across time, which is not the case. Thus, this term, and the whole title which is bit too long, should maybe be reconsidered.

We agree; the terms and the title have been modified accordingly.

Introduction:

"The specificity of the receptor of each single T cell is restricted to a small set of amino acid patterns": Any references in support of this statement? To my knowledge, a T-cell can recognize up to 106 different pMHC complexes (Mason, 1998; Wooldridge et al., 2012). Moreover, the TCR can induce structural shifts allowing the recognition of different peptides as it is the case for the BM3.3 TCR (Archbold et al., 2009; Borbulevych et al., 2009, Reiser et al., 2003).

We agree with the reviewer and removed this sentence.

Methods:

1- Additional precisions on the MiXCR parameters used to align the datasets should be provided.

This is an important aspect; we used the graphical user interface ClonoCalc and the standard parameters therein. An additional reference is now added.

2- Was the down-sampling performed using the number of sequencing reads as a threshold and thus applied on the lists of reads pre-alignment? I think this is an important aspect to be clarified

as in this case, the sampling threshold would take into account sequencing errors and unproductive sequences that might lead to an overestimation of the repertoire sizes.
The down-sampling of the 7d group was performed after removing nonfunctional nucleotide sequences. Thus, the final result of the down-sampling step is not affected by these sequences. The manuscript has been modified accordingly.
Along with this point, the down-sampling applied on the repertoires 7d post-immunization could be causing the elimination of rare clonotypes that could otherwise be contributing to the immune response, particularly as significant perturbations were observed in the CNlow fraction at this timepoint.
The reviewer is right, the down-sampling step can cause the elimination of rare clonotypes and therewith a loss of information. Nevertheless, this step is necessary because otherwise the fractions in Figure 1 and 2 would not be comparable.

3- "Each clonotype was assigned the V- and J- segments of the underlying nucleotide sequence of highest read count". Why did the authors opt for this strategy? I think assigning the most expressed VJ combination to each "set of clonotypes" might overshadow other highly expressed combinations (was the VJ distribution looked at beforehand?) subsequently giving a higher importance to certain combinations in the RHIVJ analysis.
Please see below (point 4-).

4- If I understood well, the repertoire fractioning was applied without taking into account the VJ genes. While I totally agree with the rationale of focusing on the CDR3 region while searching for relevant CDR3s in the context of an immunization in view of its center role in antigen recognition, taking into account the V-J usage in the identification of repertoire fractions could be important particularly as it is one of the two criteria on which this study is based.
To assign exactly one V- and J-segment to each clonotype is a distinct simplification which does not fully account the diversity of the T-cell receptor repertoire. We decided to perform the splitting procedure based on CDR3 sequences (i.e. clonotypes) because the specificity of the T-cell is mainly determined by this part of the receptor. The direct comparison of RHI_{VJ} and RHI_{LD} which is provided in Figure 2 requires equal partitions of the repertoire for both indices.

5- It is mentioned that the number of clonotypes ranged from 50k to 110k per sample, however these numbers do not match with the ones plotted in Fig1A. A summary table of the number of sequences/clonotypes would be much clearer.
The value 110k is indeed incorrect, we apologize for this. The number of extracted clonotypes ranges from about 50k (exactly 47,433) to about 100k (exactly 96,852). We agree that it is difficult to read off the exact values in Figure 1A due to the inevitable logarithmic scaling. Therefore, we provide an additional table with the exact values as supplemental material according to the reviewers suggestion.

Figures:
Fig. 1:
1- Title: The use of the term "SRBC-specific" is not appropriate as no functional assays were performed to validate the specificity of the identified clones.
We agree and changed it throughout the manuscript.

2- Fig1B: an "e" is missing in "sequence"
Thanks.

3- Was the arbitrary fractioning completely random or based on a certain percentage of the top clonotypes? I am curious about the number of clones being that homogeneous between experimental groups for the CNlow and CNmed but not the CNhigh fractions.

Our definition CNhigh ensures that this fraction contains on average about 100 clonotypes (exactly 119). Therefore, this fraction is comparable to the analysis of the top 100 clonotypes with the highest copy number which is performed in many studies. We added a further explanation and references to the manuscript. The inhomogeneity in the CNhigh fraction can be explained by the relatively low number of clonotypes in this fraction. In addition, the effect is visually enhanced by the logarithmic scale.

4- What does "clonotype" refer to in this section? If I refer to the materials and methods, a clonotype is a "set of T cells with identical CDRβ region", and thus does not include the V and J genes. With that being said, "two clonotypes are defined as similar if the LvD of their CDR regions is at maximum 1", how could two clonotypes be completely similar and thus have a LvD=0 if the VJ genes are not accounted? Shouldn't they be considered as one clonotype in this case? More precision on the definition of a clonotype is needed in this section to clarify these subtilties.

These are important considerations which were stated in "Material and Methods". We now provide a brief summary also in the results.

5- I would not consider that the RHIVJ can capture" sequence similarity", but rather the diversity in the gene usage. Two clonotypes expressing the same VJ combination but having completely different CDR3 sequences cannot be considered as similar, particularly when the specificity is mostly encoded by the CDR3 sequence.

We agree and changed it throughout the manuscript.

6- A visualization of the LvD clusters as a complement to the RHILD could bring additional information on the size/density of the formed clusters and the effect of the sequence similarity reduction observed 3d post-immunization compared to the PBS group on the cluster's architecture. Considering that private clones displace public ones (as stated in the discussion), big dense clusters should be fractioned into multiple small clusters.

This is an interesting idea, but the immunization effects to the cluster structure are too subtle to be detected visually (see below). This fits nicely to the minor effects of the immunization captured by RHI_{LD}*. We think, however, that this figure should not be included in the manuscript since it makes no additional point.*

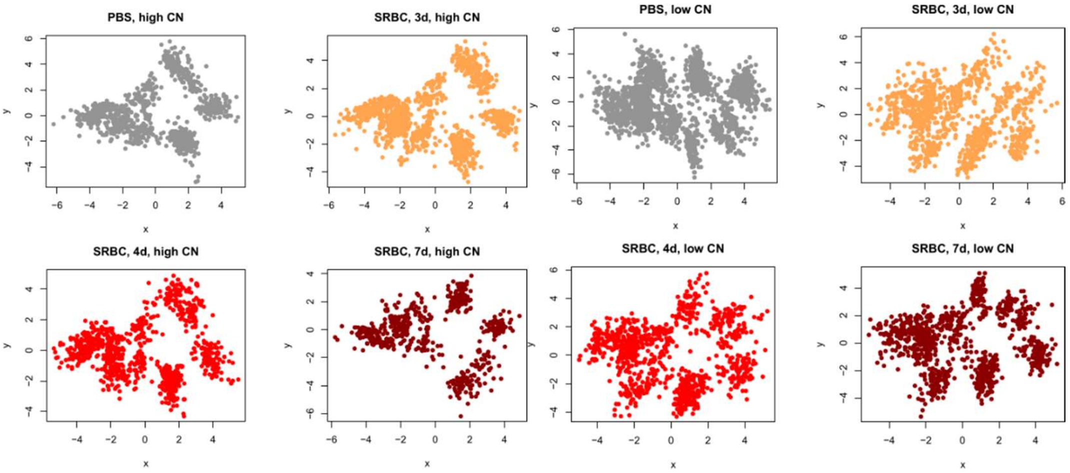

7- The use of the term "homogenization" in reference to a decrease in the number of nucleotide CDR3 sequences encoding for the same amino acid sequence is confusing, as by homogenous one would think of a homogeneous number of nucleotide sequences across all amino acid clonotypes in the repertoire. It is however not the case herein.
We agree and changed it throughout the manuscript.

Fig 2:
1- It would be easier to follow through the results if the main text and the figures have the same CN nomenclature (either use 1-9 or 2-512).
We agree and changed it throughout the manuscript.

2- It would be interesting to look at the Jaccard scores between the CNhigh fraction at day 3 and CNlow at day 7 to track whether the potentially antigen-related expansions are the ones that are detected at low frequency at a later timepoint post-immunization.
This is an interesting idea, but it can only work if a relevant fraction of the expanding clonotypes is of public nature. For each animal we have a "snapshot in time" of the receptor repertoire for exactly on time point. Thus, a clonal overlap of the expanding (and re-immigrating) clonotypes (which is measured by the Jaccard-index) would imply that clonotypes have been detected in several animals (and are therefore of public nature via definition). Therefore, the clonal overlap of SRBC 3d samples (high CN) and SRBC 7d samples (low CN) does not change if the 7d samples are replaced by the control group (see below; the error bars in the plot display mean and standard deviation).

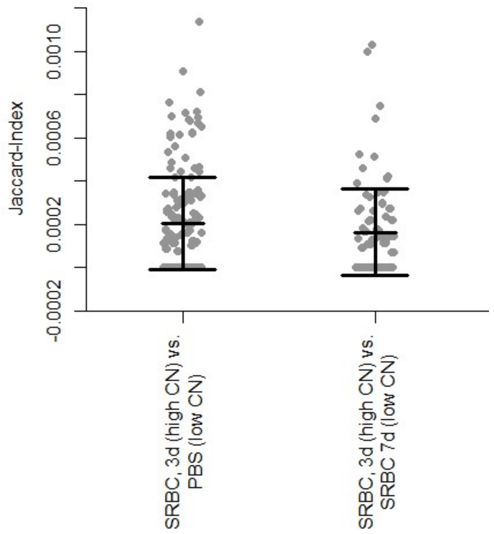

3- I'm curious about the differences observed in the 64<cn. Moreover, as RHIVJ shows significant differences for fractions with CN>64, I would assume that the perturbation in the VJ usage is not directly linked or caused by the potential SRBC-related clonotypes that are expanded 3-4 days post-immunization and which are observed within the CN>256 fraction. This might be caused by the V-J assigning strategy.
The assigning strategy has been applied to each of the experimental groups (including the control group). Thus, differences between immunized animals and the control group (which are visualized Figure 2B) cannot be caused by the assigning strategy. In addition, we observed a coherent dynamic in the fractions of 64<CN<=256. Three days after antigen exposition only moderate immunization effects were seen (0.01<p<0.05) which became highly significant at day 4.

4- The superior discriminatory power of RHIVJ compared to RHILD and CDI can be a reflection of the differential contribution of the CD8 and CD4 populations in the immune response against SRBC as the study was done on unsorted T cells. Has the CD8/CD4 ratio been looked at?
The CD8/CD4 ratio was not analyzed explicitly. Since the antigen epitopes are mainly presented by antigen presenting cells on MHC class II, it can be assumed that the response is dominated by CD4 positive T cells. Thus, the displacement of the naïve TCR-R (including CD8 positive T cells) by the expanding cells is presumably associated with a decrease of the CD8/CD4 ratio.

Fig 3:
1- Precisions on the methods used for both unsupervised and supervised classification within the main text are needed to further clarify what was done in this section.
We agree and the text in "Results" and "Material and Methods" were specified accordingly.

2- **The choice of the "relevant" fractions is arbitrary in my sense and could be the reason behind the discordant results obtained on both fractions when looking at the VJ usage and nucleotide coding. In fig2 B, significant p values are obtained with CDI on the CN< 4 and CN>256 fractions whereas significant differences are observed with RHIVJ on CN<16 and CN>64. This could explain why VJ usage but not nucleotide coding performs well on the chosen Xtop fraction (CN>64), whereas nucleotide coding classifies well on the Xbottom (CN<4) fraction. Thus, I think the choice of fractions should take into account the results obtained for each index in fig2.**
The choice of the relevant fractions was performed based on an exact criterion: fractions that reached significance either at least at two different time points or for two of the three indices. Yes, defining Xtop as the set of clonotypes with CN>256 leads to a distinct improvement of the classification result based on the nucleotide criterion. In particular the repertoires of the 3d and 4d group can be distinguished from naïve with satisfying accuracy. Nevertheless, the classification is at best moderate (half of the 7d group is misclassified, see below). This is now mentioned in the text.

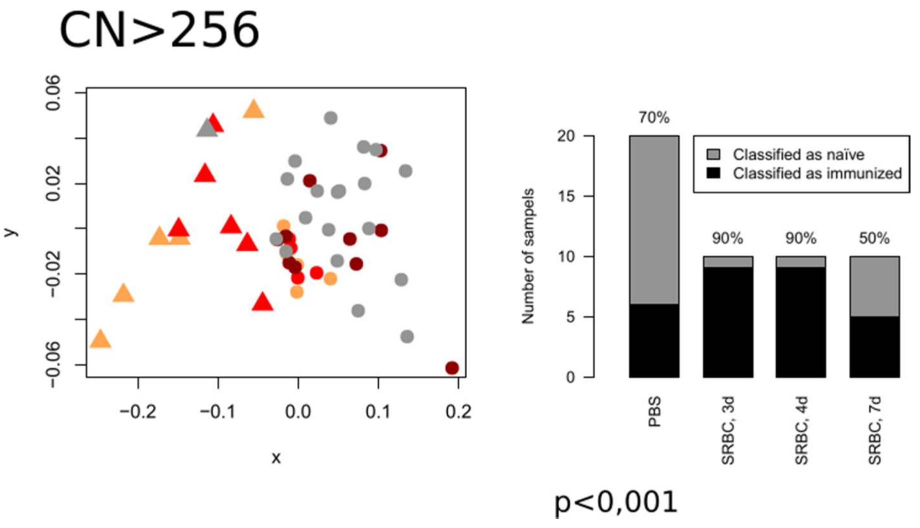

3- For the Xbottom fraction, 3d and 4d post-immunization repertoires could be used as a control in the supervised classification analysis by comparing the repertoire of each timepoint to the control group. Based on the results in fig2B, values should be similar to the ones obtained when applying random labeling.

In fact, for the Xbottom fraction the 3d and 4d post-immunization repertoires are indistinguishable from naïve. We do not show the supervised classification, because the result can be read off from the scatterplot of the cluster analysis (see below).

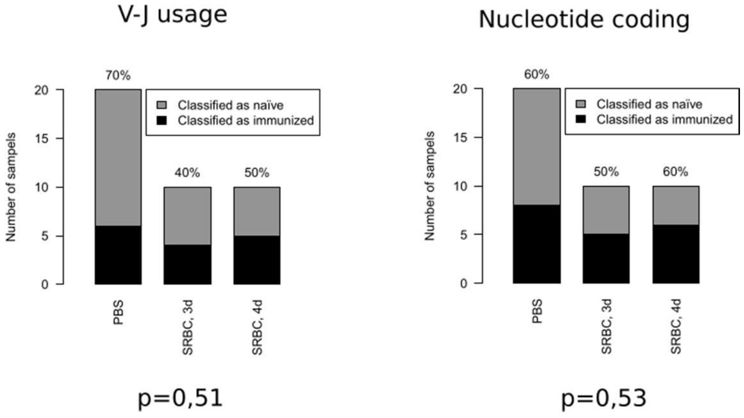

4- FigS2: Did the authors perform the classification analysis using the nucleotide coding criteria on the total repertoire?

If the nucleotide criterion is applied, only the samples of the 7d group are slightly separated from the remaining sample, displaying a similar pattern as for Xbottom. If all experimental settings are included, most samples are misclassified. After removing the 3d and 4d data, the 7d the result strips out a random labeling, but the accuracy is at best moderate. Despite from the non-satisfying classification result, these observations can be explained by the huge quantity of clonotypes in the Xbottom fraction. Thus, they do not provide any additional biological information. Nevertheless, this point is now briefly summarized in the text.

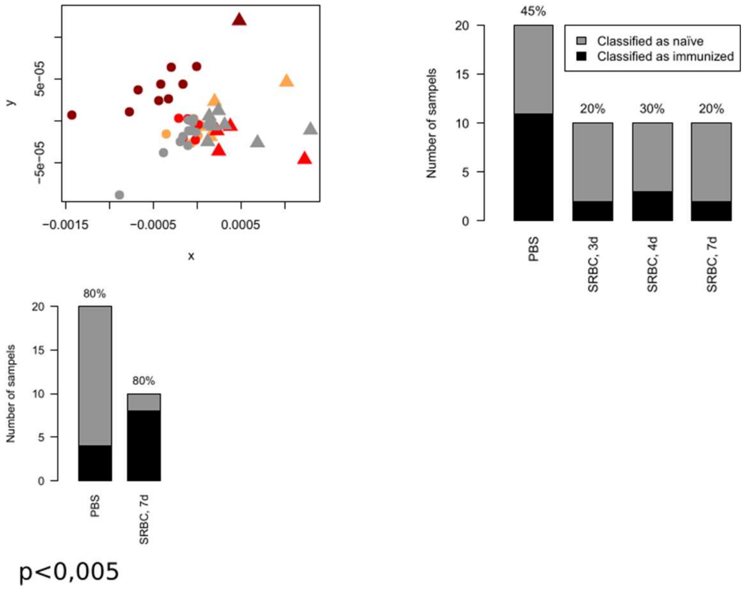

Fig4:

1- A brief description of the identification strategy of the "public SRBC-specific" clonotypes is needed in the results section. Was the strategy timepoint-dependent, i.e. applied on each timepoint compared to the control group?

We added further explanations to "Results" and "Material and Methods". A clonotype is selected if the null hypothesis that the experimental conditions (SRBC-application, timepoint of immunization) do not affect the distribution of the (random) copy number. Thus, significant effects in only one immunized group are sufficient for selection. Clonotypes which were under-expressed in all of the immunized groups were excluded from further analysis.

2- Does this strategy take into account the CN of the clonotypes across mice within the same experimental group? For example, a clonotype identified as significantly more present in the immunized group but in totally different CN fractions across the mice (CNlow and CNhigh) might not have the same weight nor contribution in the SRBC response than other clonotypes that are found within the CNhigh fraction in all mice.

We selected exactly those clonotypes whose CN distribution is significantly affected by the experimental conditions (see additional text in "Material and Methods"). The fractioning approach was not included in the differential gene expression analysis.

3- In view of the low Jaccard scores shown in fig1, I find it surprising that only 40 clonotypes were found to be enriched in the immunized group. It would maybe be interesting (if it is not already the case) to look at each time point independently as their behavior is not similar across all previous analyses.

In the differential gene expression analysis only those clonotypes were included, which were detected in at least 75% of the immunized animals (so they are public via definition). The public component represents only a miniscule minority of the total T-cell response. Thus, the decrease of the Jaccard-Index is caused mainly by private clonotypes (the expansion of public clonotypes would lead to an increasing clonal overlap).

4- Again, the term "specific public clones" is not appropriate as the specificity was not tested by functional assays.

Although the specificity of the expanding clonotypes was not tested explicitly, the observed enrichment after SRBC application can be seen as a hint that they react to an epitope derived from the injected SRBC, thus they can be seen as a public component of the SRBC induced T-cell response The text has been modified accordingly.

5- I do not think that the observations in fig 2 and 3 reflect the behavior of "private clonotypes" exclusively as no prior filtering was applied to select such clonotypes as it is the case in fig 4 for the public ones. A more appropriate comparison could be performed between the identified 40 public clones and a list of CDR3s with no significant enrichment in the immunized group in order to confirm the accumulation of the public clones within the highest fraction.

The fact that the expanding clonotypes are for the main part of private nature can be verified using the Jaccard index. If the clonal overlap of two samples is calculated and one of them taken from an animal immunized with SRBCs, the result is nearly independent from the immunization status of the second sample (see reference (1)). If the response contained a relevant quantity of reacting public clonotypes the clonal overlap between two immunized samples would be increased in comparison to an immunized and a naïve sample (because the clonal overlap would also include the reacting public clonotypes). This was observed for a T-cell response against killed Mycobacterium tuberculosis (2), indicating a relevant public component. In addition, the small number of public clonotypes which were significantly enriched after SRBC-application confirms the private nature of the T-cell response.

6- The inclusion of the RHIVJ results from fig2 is misleading and seems unnecessary in this analysis, particularly as the number of clonotypes and the RHIVJ did not show concordant observations in fig 2. It would be more interesting to look at the RHILD of the 40 public clonotypes compared to a list of non-enriched CDR3s. Furthermore, the search for amino acid motifs that are shared by the private clonotypes could reveal their potential implication in the SRBC response, or on the contrary reveal their rather bystander activation.

The color band has been removed. We agree that is confusing and unnecessary.

Reviewer 2

#1 The authors reported a rapid increase of CNhigh clonotypes at day 3 but this quickly vanished at day 4&7 (Fig 1A). Since the half-life of activated cytotoxic T cells are around 1.7 days (RJ De Boer et al, J Immunol, 2003), it is unlikely that these CNhigh clones died at this speed. Have the authors checked other organs in terms of T cell distribution?
The epitopes of the injected SRBC are mainly presented by antigen presenting cells on MHC class II complexes. Thus, it can be assumed that the response clearly dominated by CD4 positive T cells, whereas cytotoxic cells are of less importance in this mouse model. The half-life of 1.7 days was observed in the "concentration phase" of the immune reaction which starts about 8 days after antigen exposure. Since we observed the immune response only for 7 days, all observations can be ascribed to the "expansion phase" (3).

#2 The authors showed a significant change in the mean sequence length of CDR3β between day 0 and day 3. Is this a result of one specific clone/clones that have longer CDR3 regions?
In general, clonotypes of public nature display a slightly shorter CDR3-region than private ones (4). The expansion of a variety of SRBC-specific private leads to a displacement of naïve public clonotypes. This is accompanied by a slight enlargement of the CDR3 region.

#3 The authors demonstrated the dynamic changes of T cell populations with a variety of copy numbers (Fig 4). While the statistical significance of the results is appreciated, did any of the reported SRBC-specific TCR sequences appear in the sequencing repertoire?
The expanded clonotypes have been extracted from the sequencing repertoire using a differential gene expression analysis (each clonotype has been detected in at least 75% of the immunized animals). This is now described in more detail.

#4 The p values of RHJVJ were included in Fig 4, which were previously shown Fig 2B. The way Fig 4 presented is not self-explanatory. One might confuse the color bar as the statistical significance between PBS and immunized group. The authors should consider alternatives for showing the information about RHJVJ.
The color band has been removed. We agree that is confusing and unnecessary.

1. Tune C, Meinhardt M, Kalies K, Pagel R, Schierloh LK, Hahn J, u.a. Effects of sleep on the splenic milieu in mice and the T cell receptor repertoire recruited into a T cell dependent B cell response. Brain, Behavior, & Immunity - Health. 1. Mai 2020;5:100082.
2. Thomas N, Best K, Cinelli M, Reich-Zeliger S, Gal H, Shifrut E, u.a. Tracking global changes induced in the CD4 T-cell receptor repertoire by immunization with a complex antigen using short stretches of CDR3 protein sequence. Bioinformatics. 15. November 2014;30(22):3181–8.
3. Boer RJD, Homann D, Perelson AS. Different Dynamics of CD4+ and CD8+ T Cell Responses During and After Acute Lymphocytic Choriomeningitis Virus Infection. The Journal of Immunology. 15. Oktober 2003;171(8):3928–35.
4. Madi A, Shifrut E, Reich-Zeliger S, Gal H, Best K, Ndifon W, u.a. T-cell receptor repertoires share a restricted set of public and abundant CDR3 sequences that are associated with self-related immunity. Genome Res. Oktober 2014;24(10):1603–12.

We are convinced that the manuscript has profited from the comments of the reviewers, and we hope that it can now be accepted for publication in "PLOS ONE".

Yours sincerely,

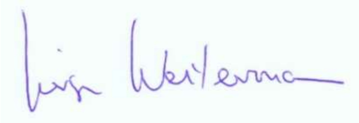

(Prof. Dr. Jürgen Westermann)
