# Supplementary material for: The splenic T cell receptor repertoire during an immune response against a complex antigen: Expanding private clones accumulate in the high and low copy number region
Source: PLoS One. 2022 Aug 24;17(8):e0273264. doi: 10.1371/journal.pone.0273264 (PMC9401120; doi:10.1371/journal.pone.0273264)
Supplement: Attachment — Submitted filename: 20220603 PLOS One ReviewCorrected.docx [file pone.0273264.s005.docx]

**Summary**

In this manuscript, Meinhardt et al. reported a novel approach to estimate the diversity of TCRs using CDR3β and VJ usage. The authors utilize this model to reveal the T cell repertoire dynamics post-immunization. This approach can potentially be also used in studying a variety of immune responses. The manuscript is suitable for publishing in PLOS ONE.

**Comments for the authors to consider:**

**#1** The authors reported a rapid increase of CN^high^ clonotypes at day 3 but this quickly vanished at day 4&7 (Fig 1A). Since the half-life of activated cytotoxic T cells are around 1.7 days (RJ De Boer et al, J Immunol, 2003), it is unlikely that these CN^high^ clones died at this speed. Have the authors checked other organs in terms of T cell distribution?

**#2** The authors showed a significant change in the mean sequence length of CDR3β between day 0 and day 3. Is this a result of one specific clone/clones that have longer CDR3 regions?

**#3** The authors demonstrated the dynamic changes of T cell populations with a variety of copy numbers (Fig 4). While the statistical significance of the results is appreciated, did any of the reported SRBC-specific TCR sequences appear in the sequencing repertoire?

**#4** The p values of RHJ_VJ_ were included in Fig 4, which were previously shown Fig 2B. The way Fig 4 presented is not self-explanatory. One might confuse the color bar as the statistical significance between PBS and immunized group. The authors should consider alternatives for showing the information about RHJ_VJ_.
