# Supplementary material for: The splenic T cell receptor repertoire during an immune response against a complex antigen: Expanding private clones accumulate in the high and low copy number region
Source: PLoS One. 2022 Aug 24;17(8):e0273264. doi: 10.1371/journal.pone.0273264 (PMC9401120; doi:10.1371/journal.pone.0273264)
Supplement: Attachment — Submitted filename: 20220802 PLOS One D-22-11828 2nd Review.docx [file pone.0273264.s007.docx]

**Summary**

In this manuscript, Meinhardt et al. utilized a new model to estimate the dynamic diversity of TCRs post-immunization. The authors have addressed some of the concerns and comments in my previous review. The revised version has improved clarity and more detailed methodology. The manuscript is suitable for publishing on PLOS ONE.

**Comments for the authors (Revision is not needed):**

I am satisfied with the authors’ responses. No further edit is needed.
